# Assessing the Value of T cell Monitoring in the General Population of Children and Adolescents – Insights From the Ciao Corona Cohort Study

**DOI:** 10.3389/ijph.2025.1608612

**Published:** 2025-09-18

**Authors:** Alessia Raineri, Sonja Rueegg, Thomas Radtke, Susi Kriemler, Kyra Zens, Giuseppe Pantaleo, Craig Fenwick, Céline Pellaton, Milo A. Puhan

**Affiliations:** ^1^ Epidemiology, Biostatistics, Prevention Institute, University of Zurich, Zurich, Switzerland; ^2^ Division of Immunology and Allergy, Lausanne University Hospital (CHUV), University of Lausanne (UNIL), Lausanne, Switzerland

**Keywords:** COVID-19, SARS-CoV-2, cellular immunity, humoral immunity, surveillance

## Abstract

**Objectives:**

Serological monitoring of SARS-CoV-2 antibodies in children and adolescents has been crucial for pandemic surveillance and response, while the role of T cell response monitoring remains uncertain. This study aimed to assess the potential of T cell response monitoring in children and adolescents during the COVID-19 pandemic.

**Methods:**

We compared antibody and T cell-mediated immune responses in a subpopulation (n = 109) of children and adolescents from the Ciao Corona cohort study. Participants were followed up over 6 months, from November/December 2021 to June/July 2022, during the Delta and Omicron waves in Switzerland.

**Results:**

Circulating, virus-specific T cell responses in children and adolescents were generally low and heterogeneous. T cell-mediated responses were not consistent with SARS-CoV-2 infection status, with responses detectable in some seronegative individuals and not detectable in a considerable number of seropositive participants.

**Conclusion:**

Circulating, virus-specific T cell responses to SARS-CoV-2 in children and adolescents from the general population offer limited additional insight. Monitoring humoral immunity is likely a more cost-effective approach and should be the primary focus of immunological surveillance in the general paediatric population.

## Introduction

In the context of pandemic surveillance and response, T cell-mediated responses may complement the well-established serological monitoring of virus- and variant-specific antibodies in the general population of children and adolescents. During the COVID-19 pandemic, numerous studies assessed humoral immunity in children and adolescents and provided valuable insights into their immune responses to SARS-CoV-2 infections and vaccinations [1–5]. These studies yielded consistent and actionable results that have informed public health strategies.

T cell-mediated responses are, unlike antibodies, less commonly studied, especially in the general population of children and adolescents [6–8]. T cells play an important role in controlling viral infections by targeting and eliminating infected cells, and understanding their dynamics is crucial for a comprehensive view of immune protection [9–11]. Very few studies have assessed T cell-mediated responses to SARS-CoV-2, often including subpopulations with small sample size, ranging from asymptomatic and/or mildly symptomatic to hospitalized children and adolescents, or those affected by multisystem inflammatory syndrome (MIS-C) [12–21]. The results from these studies have been highly heterogeneous, making it difficult to draw broad conclusions about the utility of monitoring T cell responses in the general paediatric population. Thus, the unique conditions of the pandemic, with widespread infections, vaccinations, and varied exposure histories, provide an ideal setting to study T cell-mediated responses in this population.

While we previously presented detailed results on antibody trajectories during the pandemic [22], here we aimed to assess the surveillance potential of T cell responses in children and adolescents during the COVID-19 pandemic. We followed up seropositive and seronegative children and adolescents from a school-based study (Ciao Corona) over 6 months (i.e., November/December 2021 to June/July 2022), comparing their cellular and humoral immune responses.

## Methods

### Study Setting and Design

The Ciao Corona study is part of the nationally coordinated research network *Corona Immunitas* [23] in Switzerland. The study protocol was published prospectively (ClinicalTrials.gov identifier: NCT04448717) [24] and the seroprevalence results of all testing rounds can be found elsewhere [22, 25–28]. This cellular immune response analysis is based on a subset of children and adolescents who participated in the testing rounds of either November/December 2021, June/July 2022, or both testing rounds within the Ciao Corona sample. The study was conducted in the canton of Zurich, which has a population of 1.52 million people. This represents 18% of the Swiss population and includes residents from diverse ethnic and linguistic backgrounds, encompassing inhabitants from both rural and urban areas. The study was approved by the Ethics Committee of the Canton of Zurich, Switzerland (Registration No. 2020-01336). Prior to the study enrolment, children and adolescents provided verbal and parents/proxies provided written informed consent.

### Population

In Ciao Corona, we included randomly selected primary schools from the canton of Zurich and matched the nearest geographically located secondary school. We invited schools based on the population size of the 12 districts. We contacted 156 schools to take part in the study, and a total of 55 schools, 54 public and 1 private, decided to participate. Classes were stratified by school level and then randomly selected, encompassing grades 1–2 (children aged 6–8) at the lower school level, grades 4–5 (children aged 9–11) at the middle school level, and grades 7–9 (adolescents aged 12–14) at the upper school level. All children and adolescents from the selected classes were eligible to participate at any point in the study. We conducted five testing rounds between Jun/Jul 2020 and Jun/Jul 2022 and collected venous blood samples for antibody detection (anti-spike IgG and anti-nucleocapsid IgG). The first testing round (T1) took place in June/July 2020, the second (T2) in October/November 2020, the third (T3) in March/April 2021, the fourth (T4) in November/December 2021 and the last fifth (T5) testing round in June/July 2022.

In Nov/Dec 2021 we selected a convenience-based subsample of children and adolescents (N total = 109) for analysis of cellular immune responses, limiting sampling to the first five children and adolescents meeting the inclusion criteria per testing day due to limited resources. We invited children and adolescents that previously participated in at least one, but preferably two consecutive testing rounds (i.e., T1-T3). We included unvaccinated seropositive and seronegative children and adolescents in November/December 2021 and followed them up in June/July 2022, regardless of their vaccination status. For this subsample we collected additional venous blood for Interferon (IFN)-gamma ELISpot (N = 109) and IFN-gamma Release Assay (IGRA) (N = 26, randomly selected)-based analyses.

### Categorization of Children and Adolescents Based on Their Exposure Status and Seropositivity

Children and adolescents from the longitudinal cohort were divided into four different groups based on their vaccination and infection history to analyse the evolution of anti-spike IgG and neutralizing antibody levels over time. Adolescents aged 16 and older were eligible for vaccination starting in May 2021, those aged 12 to 15 became eligible in mid-June 2021, and children aged 5 to 11 were eligible starting in January 2022 [29]. Individuals who never tested positive for anti-spike IgG were labelled as *negative* and unvaccinated children and adolescents who tested positive for anti-spike IgG at any study point as *infected*. Individuals who were vaccinated but always tested negative for anti-spike IgG prior to vaccination and afterwards never tested positive for anti-nucleocapsid IgG were categorized as *vaccinated*. Finally, those who were seropositive before vaccination, or those who were vaccinated and tested positive for anti-nucleocapsid IgG, were classified as *hybrid*.

### Isolation of Plasma and PBMCs

In all testing rounds venous blood samples were collected and centrifuged to isolate plasma. Plasma aliquots were stored at −20 °C prior to antibody analyses. To isolate peripheral blood monocytic cells (PBMCs), blood samples were subjected to density-gradient centrifugation using Ficoll–Paque (density 1.077 g/mL). PBMCs were frozen in freezing media (90% fetal bovine serum (FBS, Pan Biotech) mixed with 10% dimethyl sulfoxide (DMSO, Sigma)) at −80 °C and subsequently transferred to liquid nitrogen prior to use in the ELISpot assay.

### Serology

We employed the Sensitive Anti-SARS-CoV-2 spike Trimer Immunoglobulin Serological (SenASTrIS) test to detect SARS-CoV-2-specific antibodies targeting spike and nucleocapsid proteins. The Bio-Plex 356 Amine Coupling Kit (Bio-Rad, Catalogue 10000148774) was used to covalently couple MagPlex beads to either the SARS-CoV-2 spike protein trimer or nucleocapsid protein following the manufacturer’s instructions. Diluted protein-coupled beads were added to each well of the flat bottom 96-well plates (Bio-Plex Pro, Bio-Rad) and washed with PBS using a magnetic plate washer (MAG2x programme).

Next, 50 μL of each plasma sample was diluted 1:300 in PBS and added to the wells. As negative control, a pool of pre-pandemic healthy human serum was utilized (BioWest human serum AB males; VWR). The plates were incubated for 1 h at room temperature with shaking, followed by PBS washes, and then incubated with 50 μL of a 1:100 dilution of polyclonal Goat F(ab’)2 anti-human IgA-PE (for the anti-IgA assay; Southern Biotech; Catalogue 2052-09) or polyclonal Goat anti-human IgG-PE (for the anti-IgG assay; OneLambda; Catalogue LS-AB2) as a secondary antibody for additional 45 min at room temperature while shaking. Following incubation, the samples were washed with PBS, resuspended in reading buffer, and mean fluorescence intensity (MFI) values were measured for each sample using a Bio-Plex (Luminex) 200 plate reader with the Bio-Plex Manager software (version 6.2; Bio-Rad). The MFI ratio was calculated by dividing the MFI value of each plasma sample by the mean value of the negative control samples. For both anti-spike IgG and anti-nucleocapsid IgG antibodies, samples were considered seropositive if their MFI ratios were equal to or exceeded the cutoff value of 6, corresponding to the assay’s 99.2% specificity and 94.0% sensitivity. The cutoff of 6 was established based on results from negative control samples and from SARS-CoV-2 PCR-positive individuals. As the assays used in this study demonstrated stable performance with only minimal decay over a period of up to 8 months post-infection, we did not account for sensitivity changes due to time-related decay.

### ELISpot Assay

T cell-mediated responses of our subsample were evaluated as described previously [30, 31] by IFN-gamma ELISpot (R&D Systems, Catalog EL285). In brief, cryopreserved PBMC samples were thawed and seeded at density of 5e5 cells per well. Cells were stimulated for 20 h at 37 °C and 5% CO2 with 15mer peptide pools covering the entire amino acid sequences of viral M or N proteins, the S1 domain from the spike protein, or a combination of the predicted immunodominant peptides of the spike protein of which most belong to the S2 domain (M, N, S1, and S PepTivator peptide pools, respectively; Miltenyi Biotec). Peptides were used at final concentration of 0.6 nmol (∼1 μg/mL) per peptide. As a negative control, unstimulated cells were incubated in culture medium alone and as a positive control, 2.5e5 cells per well were stimulated with 10 μg/mL anti-CD3 antibody (Clone OKT3, Miltenyi Biotec, Catalog 130–093-387). Spot counts were determined using an AID iSpot Reader System and analyzed with EliSpot 7.0 software (AID). Spot numbers in negative control wells were doubled and subtracted from test wells with negative counts set to zero. Results were expressed as spot-forming units (SFU) per 1e6 PBMCs.

### Interferon-Gamma-Release Assay (IGRA)

From a subsample of 26 children and adolescents sampled in Jun/Jul 2022, T cell responses were further characterized by IGRA as described previously [31]. Briefly, whole blood was collected in Li-heparin vacutainer tubes (BD) and 250 uL per well plated in 96 well U-bottom plates (Sarstedt). Samples were stimulated with combined pools of M and N (M/N) peptides or S1 and S (S1/S2) peptides at a final concentration of 0.6 nmol (∼1 μg/mL) per peptide and incubated for 20 h at 37 °C and 5% CO2. As a negative control, unstimulated blood, incubated without peptide was included and, as a positive control blood was stimulated with 10 mg/mL anti-CD3 antibody. After incubation, stimulated supernatants were stored at −20 °C prior to analysis of IFN-gamma levels by ELISA assay (Human IFN-gamma DuoSet ELISA kit, R&D Systems, Catalog DY285B, and DuoSet ELISA Ancillary Reagent Kit 2, R&D Systems, Catalog DY008), following the manufacturer’s instructions. Samples were measured using a Multiskan SkyHigh Microplate Spectrophotometer with SkanIt software (Thermo Fisher Scientific) set to a wavelength of 450 nm with a 540 nm correction and IFN-gamma concentrations calculated from absorbance values by applying a 4 PL standard curve. Concentrations from the unstimulated negative controls were subtracted from each test well with negative values adjusted to zero and expressed as IFN-gamma in pg/mL. Results were excluded if background IFN-gamma concentrations in unstimulated control wells exceeded five times the median of the test wells or if values of anti-CD3-stimulated positive controls were negative.

## Results

Table 1 shows the basic demographic and humoral immune characteristics of the longitudinal cohort at baseline (November/December 2021) and follow-up (June/July 2022). At baseline, 61% of the participants were seropositive for anti-spike IgG with 18% also seropositive for anti-nucleocapsid IgG, indicating a recent infection. At follow up, in contrast, 99% were seropositive for anti-spike IgG with 77% of them also seropositive for anti-nucleocapsid IgG. Between baseline and follow up testing, 26 of the 109 children and adolescents were vaccinated. Based on SARS-CoV-2 anti-spike and anti-nucleocapsid IgG antibody profile and vaccination history at each timepoint we stratified participants into four categories: hybrid immunity, vaccinated, infected, and negative (Table 2).

**TABLE 1:** Baseline characteristics of the longitudinal study population at baseline (November/December 2021) and follow-up (June/July 2022) (Switzerland. 2025).

	Baseline November/December 2021	Follow-Up June/July 2022
Participants, n	109	109
Sex (male), n (%)	52 (48%)	52 (48%)
Age range, years	12 (8–17)	12 (9–17)
<12, n (%)	68 (62%)	51 (47%)
≥12, n (%)	41 (38%)	58 (53%)
Chronic Health Conditions^a^, n (%)	30 (28%)	27 (25%)
Vaccinated, n (%)	0 (0)	26 (24%)
Anti-S-IgG pos^b^, n (%)	66 (61%)	108 (99%)
Anti-S-IgG neg^b^, n (%)	43 (39%)	1 (1%)
Anti-N-IgG pos^c^, n (%)	20 (18%)	84 (77%)
Anti-N-IgG neg^c^, n (%)	89 (82%)	25 (23%)

^a^ Chronic conditions reported by parents in the questionnaire: asthma, hay fever, celiac disease, lactose intolerance, allergies (other than hay fever), neurodermatitis, diabetes mellitus, inflammatory bowel disease, hypertension, arthritis, other chronic diseases potentially affecting the immune response (neutropenia, periodic fever with aphthous stomatitis, pharyngitis, and adenitis (PFAPA) syndrome, renal failure, cystic fibrosis, bronchitis).

^b^ Anti-spike IgG antibody.

^c^ Anti-nucleocapsid IgG antibody.

**TABLE 2:** T cell positivity (specific to SARS-CoV-2 M, N, S1, and S2 epitope, detected with the ELISpot assay) according to the participants exposure status in December/November 2021 and June/July 2022 (N total = 109) (Switzerland. 2025).

Timepoint	Exposure status	N Total	M Pos^a^	N pos^a^	S1 pos^a^	S2 pos^a^
*Baseline*						
December/November 2021	Infected, N (%)	66	16 (24%)	29 (44%)	28 (42%)	25 (38%)
December/November 2021	Negative, N (%)	43	9 (21%)	12 (28%)	19 (44%)	14 (33%)
*Follow Up*						
June/July 2022	Hybrid, N (%)	19	7 (37%)	7 (37%)	8 (42%)	9 (47%)
June/July 2022	Vaccinated, N (%)	7	2 (28%)	2 (28%)	2 (28%)	4 (58%)
June/July 2022	Infected, N (%)	82	40 (48%)	43 (52%)	47 (57%)	43 (52%)
June/July 2022	Negative, N (%)	1	0	0	0	0

^a^ positive T cell-mediated response specific to SARS-CoV-2 M, N, S1 or S2 epitopes.

M, Membrane; N, Nucleocapsid; S, Spike.

**FIGURE 1:**
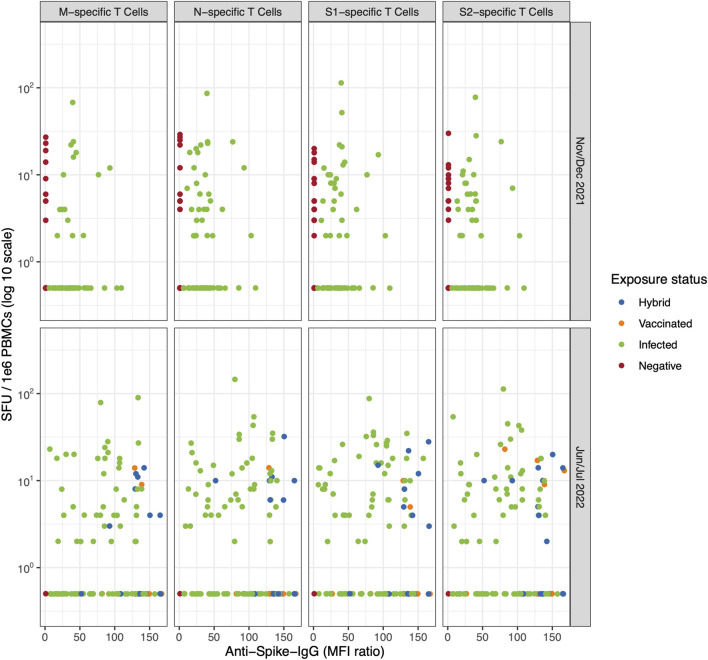
The correlation between T cell-mediated response (specific to SARS-CoV-2 M, N, S1, and S2 epitope, detected with the ELISpot assay) and anti-spike IgG response colored by participants’ exposure status (Switzerland. 2025). The evolution of T cell-mediated response, number of spot-forming units (SFU) per 1e6 peripheral blood mononuclear cells (PBMCs) following stimulation with M, N, S1, or S2 overlapping peptide pools compared to the anti-spike IgG mean fluorescence intensity (MFI) ratios at baseline (November/December 2021) and follow up (June/July 2022) in 109 participants. Children and adolescents are coloured based on their exposure status (i.e., hybrid, vaccinated, infected and negative). Table 2 shows the corresponding percentage of T cell positivity by exposure status. M, Membrane; N, Nucleocapsid; S, Spike (Switzerland. 2025).

We next assessed T cell responses by comparing baseline and follow-up blood samples. Overall, we found an increase in the frequency of individuals responsive to each of the tested peptide pools (SARS-CoV-2 M, N, S1, and S2), as well as individuals reactive to all 4 pools (baseline: 17% and follow-up: 27%) or to at least one pool (baseline: 56% and follow-up: 76%). However, responses were generally low and heterogenous (Figure 1; Table 2). To confirm the overall low levels of T cell responsiveness observed in the cohort, we also tested a subsample of 26 children and adolescents by IGRA at follow up (baseline characteristics of cohort can be found in Supplementary Table S1). We found that T cell-mediated responses were also low according to this assay (see Supplementary Figures S1, S2).

**FIGURE 2:**
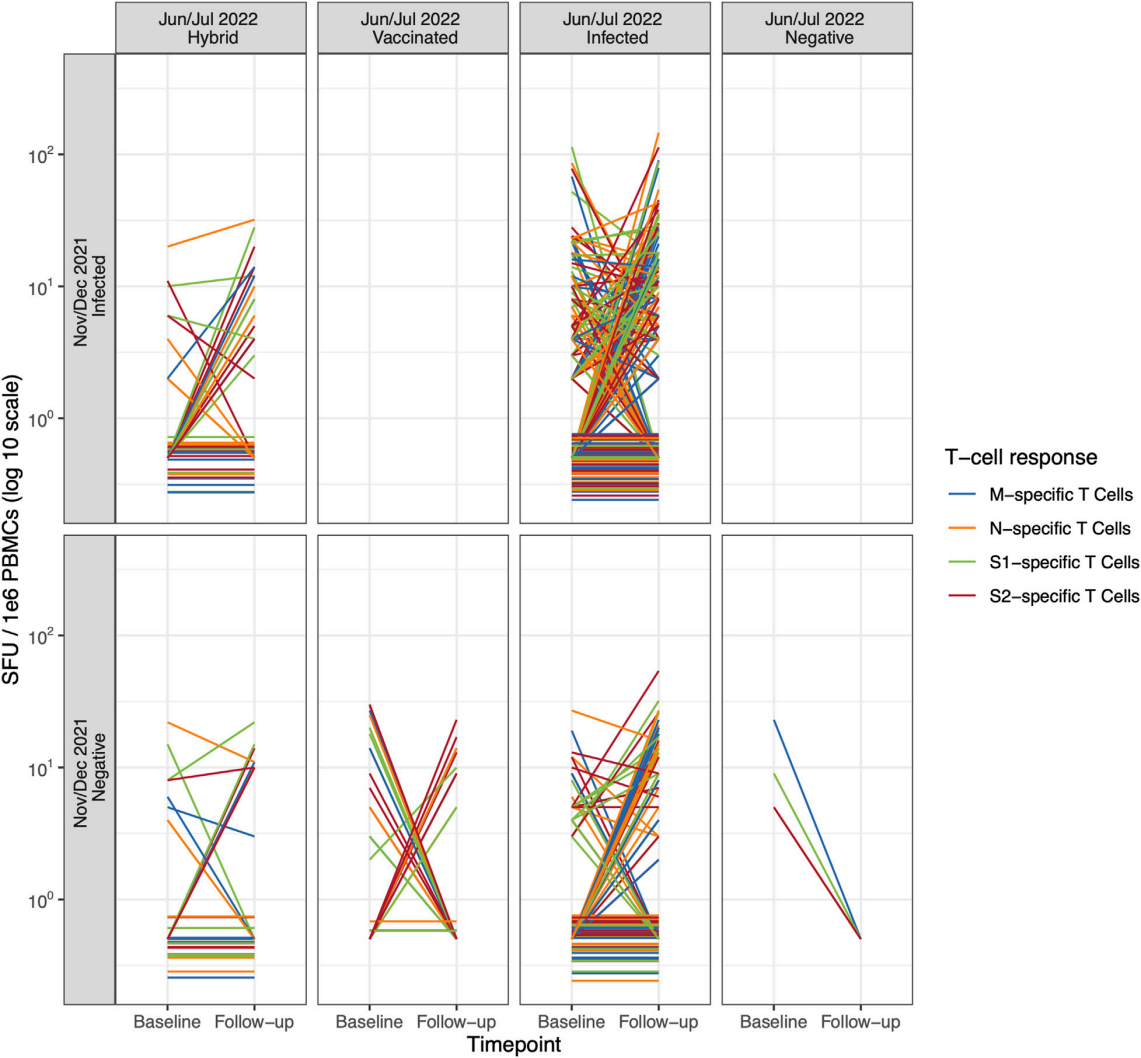
Individual trajectories of T cell-mediated response (specific to SARS-CoV-2 M, N, S1, and S2 epitope, detected with the ELISpot assay) between baseline (November/December 2021) and follow-up (June/July 2022) according to the participants’ exposure status (Switzerland. 2025). The individual trajectories of T cell-mediated response specific to SARS-CoV-2 M, N, S1, and S2 epitopes for each participant between baseline and follow-up. Children and adolescents are categorized by their individual exposure status over time. Color denotes T cell-mediated response specific to SARS-CoV-2 M, N, S1, and S2 epitopes. Note: To improve visibility, T cell-mediated responses of participants that were zero at both baseline and follow-up were scattered slightly around zero. M, Membrane; N, Nucleocapsid; S, Spike (Switzerland. 2025).

We did not observe any difference in M-, N-, S1-, and S2-specific T cell-mediated response patterns when stratifying by participant exposure status (Figure 1; Table 2), even when examining individual trajectories of T cell responses between baseline and follow-up (Figure 2; Supplementary Table S2; Supplementary Figure S3).

Furthermore, analysing participants with recent infections, as assessed by the presence of anti-nucleocapsid IgG antibodies, we did not observe differences in T cell-mediated responses over time (see Supplementary Figure S4). While, T cell responses were detectable in 56% of anti-spike and/or anti-nucleocapsid positive participants (reactive to at least one peptide-pool of SARS-CoV-2 M, N, S1, and S2) at baseline, we observed that 56% of seronegative participants also had detectable T cell-mediated responses at levels comparable to those of T cell-responsive seropositive participants.

At follow-up, only one participant remained seronegative with no detectable T cell response, whereas 63% of participants with hybrid immunity, 57% vaccinated individuals and 82% infected participants were reactive to at least one peptide-pool of SARS-CoV-2 M, N, S1, and S2.

## Discussion

In this study, we found that circulating T cell-mediated immune responses in the general population of children and adolescents are generally low, highly heterogeneous and non-specific for their SARS-CoV-2 infection status (i.e., negative, infected, vaccinated and hybrid). Notably, circulating virus-specific T cells were not readily detectable in a considerable number of seropositive participants, while such cells were observed in a subset of anti-spike IgG seronegative individuals.

Although overall T cell responses did increase in children and adolescents throughout the follow up period, we found a general lack of a clear, circulating T cell-mediated response pattern, even among individuals with antibody-confirmed exposure to SARS-CoV-2 and/or vaccination. This finding was independent of the assay used (IGRA or ELISpot). The lack of a clear T cell response stands in contrast to comparable studies in adults, where T cell-mediated responses are more robust and may better reflect exposure history [8, 14, 16, 30]. Indeed, T cell responses following both vaccination and infection, including respiratory infection, in early life are well-known to differ from those of adults and several recent studies have demonstrated that infection-specific memory T cells are likely to be initiated and accrue in the tissues, with circulating T cells being predominately naïve [32, 33]. Additionally, others have found that T cells generated during acute SARS-CoV-2 infection have a phenotype more consistent with follicular helper CD4^+^ T cells, which promote antibody production, but are not important sources of IFN-gamma, which we have evaluated here [15]. The lower SARS-CoV-2-specific T cell-mediated response in children and adolescents further suggests that other components of the immune system, particularly the innate immune response, play a more significant role in clearing the virus [34, 35].

In contrast to our findings, a recent study followed vaccinated children with repeated individual follow-ups and found that breakthrough infection after vaccination led to stronger and more diverse T cell responses, similar to features of hybrid immunity described in adults [36]. The discrepancy with our results likely reflects differences in cohort composition and study design, as our cohort predominantly consisted of unvaccinated children and adolescents and was assessed only once at a fixed timepoint, probably limiting our ability to account for the timing of immune-modifying events.

Considering that analysis of T cell responses require the isolation of PBMCs or direct stimulation of whole blood, as well as specialized laboratory techniques such as flow cytometry, ELISpot, or intracellular cytokine staining, their assessment is more time-consuming and labour-intensive. In line with this, irrespective of its clinical relevance, antibody testing is generally more straightforward and therefore, more cost-effective [31]. As shown in our recent publication and in other studies, anti-spike IgG antibody titers show a clear pattern according to the exposure status [22, 37]. Children and adolescents with hybrid immunity showed the highest SARS-CoV-2 anti-spike IgG titers, followed by vaccinated and then infected individuals [22]. Antibody surveillance can therefore serve as a practical and reliable method to monitor population-level immunity at a specific moment and over time, especially in resource-limited settings and time-limited scenarios such as the COVID-19 pandemic. Thus, surveillance efforts in younger populations may benefit more from focusing on humoral rather than cellular responses.

### Strengths and Limitations

The study focused on the general population of children and adolescents, unlike other studies that primarily included specific subpopulations, such as hospitalized children, making the findings more broadly applicable. In contrast to these studies, we had a larger sample size, which strengthens our findings about T cell-mediated responses in this population. The longitudinal study design allowed us to assess changes in both cellular and humoral immune responses over 6 months, taking into accounting for infections, vaccinations, and their combinations throughout the COVID-19 pandemic.

However, there are several limitations that need to be acknowledged. First, the exact timing of SARS-CoV-2 infections in children and adolescents was not known due to the sero-epidemiological study design. This limits our ability to account for the time to and after immune-modifying events (such as infection and/or vaccination), which may influence the strength and detectability of immune responses and thus contribute to the lack of clear correlations between measures. Second, the study relied on ELISpot and IGRA assays to assess immune responses, which, while widely used, have limitations in terms of sensitivity and comprehensiveness in capturing the full spectrum of immune activity. Third, the analysis was restricted to only four domains of the T cell-mediated response, which might not capture the entire complexity of T cell-mediated immunity, potentially overlooking other important aspects of the immune response. Finally, selection bias may be a concern due to our subsample. However, comparisons with the full sample (see recent publication [22]) showed no differences except in vaccination status, which reflects our study’s inclusion criteria. Fourth, infections in our population-based cohort were predominantly asymptomatic to mild, and as we did not collect detailed data on infection severity, it remains uncertain whether our findings can be generalized to individuals with more severe disease.

### Conclusion

In conclusion, this study indicates that circulating, virus-specific T cell responses in the general population of children and adolescents are low, heterogenous and not reflective of prior infections and vaccinations. Adding the assessment of cellular immunity to serological surveillance of the general population of children and adolescents appears to be of limited value but comes with substantial effort and cost. Thus, monitoring humoral immunity is likely a more cost-effective approach and should be the primary focus of immunological surveillance in the general paediatric population.

## Data Availability

All data used in this study are openly available and properly cited within the article. No additional datasets were generated.
