# Supplementary material for: Assessing the Value of T cell Monitoring in the General Population of Children and Adolescents – Insights From the Ciao Corona Cohort Study
Source: Int J Public Health. 2025 Sep 18;70:1608612. doi: 10.3389/ijph.2025.1608612 (PMC12489368; doi:10.3389/ijph.2025.1608612)
Supplement: Supplementary file 1 [file Supplementaryfile1.docx]

**Supplementary Materials**

**Supplementary Figure 1:** The correlation of T cell-mediated response between ELISpot and IGRA assay for T cell-mediated response specific to SARS-CoV-2 M and N. ELISpot: number of spot-forming units (SFU) per 1e6 peripheral blood mononuclear cells (PBMCs) following stimulation with M and N, overlapping peptide pools. IGRA: IFN-gamma production following M/N peptide stimulation of whole blood

IFN: Interferon; M, membrane; N, nucleocapsid

IGRA: Interferon-gamma-release assay


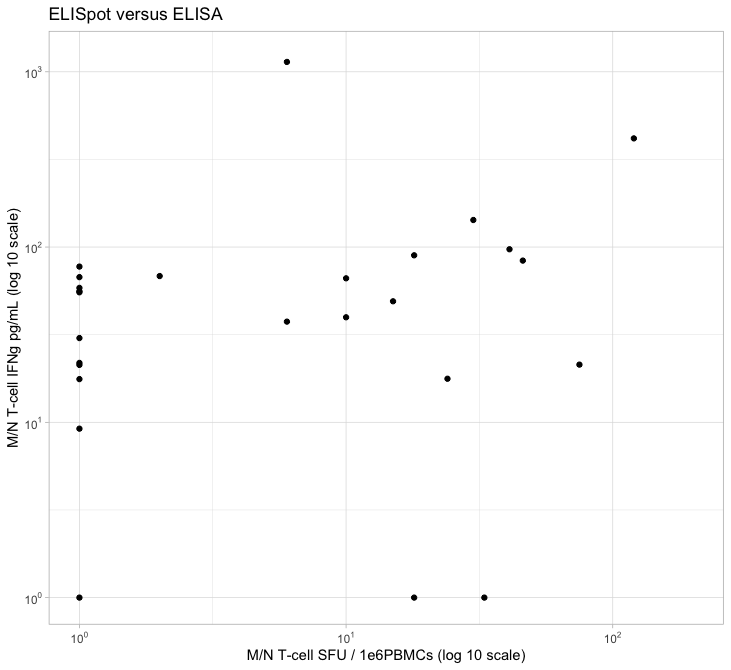


**Supplementary Figure 2:** The correlation of T cell-mediated response between ELISpot and IGRA assay for T cell-mediated response specific to SARS-CoV-2 S1 and S2. ELISpot: number of spot-forming units (SFU) per 1e6 peripheral blood mononuclear cells (PBMCs) following stimulation with S1 and S2, overlapping peptide pools. IGRA: IFN-gamma production following S1/S2 peptide stimulation of whole blood.

IFN: Interferon; S: Spike

IGRA: Interferon-gamma-release assay


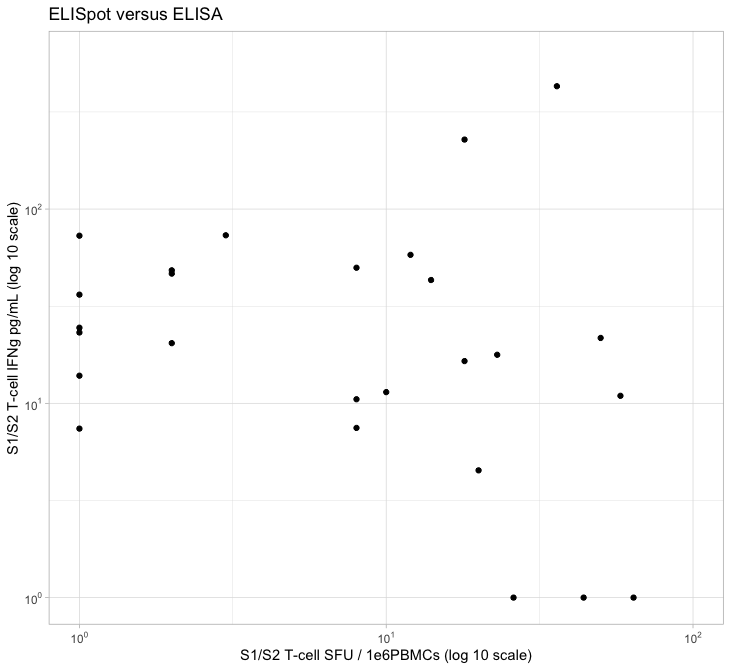


**Supplementary Figure 3:** The individual trajectories of T cell-mediated responses specific to SARS-CoV-2 M, N, S1, and S2 epitopes by ELISpot assay for each infected participant between baseline and follow-up, coloured by epitope type (M, N, S1 and S2 epitopes). Note: To improve visibility, T cell-mediated responses of participants that were zero at both baseline and follow-up were scattered slightly around zero.

M, membrane; N, nucleocapsid, S: Spike


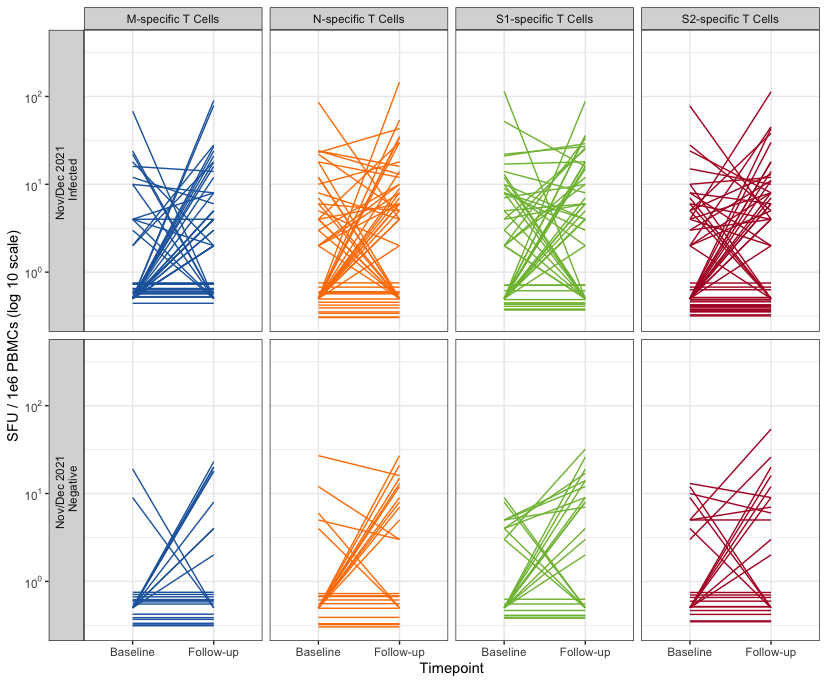


**Supplementary Figure 4:** The development of T cell-mediated response (specific to SARS-CoV-2 M, N, S1, and S2 epitope, assessed by ELISpot assay) and anti-spike IgG response categorized by participants’ exposure status.

The evolution of T cell-mediated response, number of spot-forming units (SFU) per 1e6 peripheral blood mononuclear cells (PBMCs) following stimulation with M, N, S1, or S2 overlapping peptide pools compared to the anti-spike IgG mean fluorescence intensity (MFI) ratios at baseline (November/December 2021) and follow up (June/July 2022) in 109 participants. Children and adolescents are coloured by their anti-nucleocapsid IgG antibody positivity.

M, membrane; N, nucleocapsid, S: spike


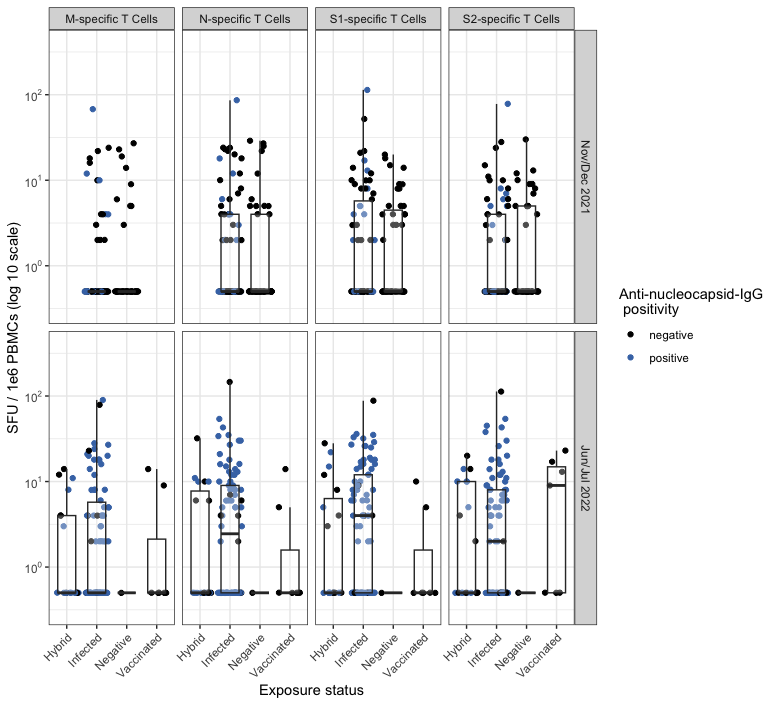


**Supplementary Table 1**: Baseline characteristics of the subpopulation (n = 26) at Jun/Jul 2022. Interferon-gamma-release assay was performed exclusively for this subpopulation.

| June / July 2022 | Cohort | Sub-Cohort |
| --- | --- | --- |
| Participants, n | 109 | 26 |
| Sex (m), % | 52 (48%) | 13 (50%) |
| Age range, years | 12 (9 - 17) | 11 (9 - 13) |
| <12, n | 51 (47%) | 18 (69%) |
| ≥12, n | 58 (53%) | 8 (31%) |
| Chronic health conditions *, n | 27 (25%) | 5 (19%) |
| Vaccinated, n | 26 (24%) | 3 (12%) |
| Anti-S-IgG pos^a^, n | 108 (99%) | 25 (96%) |
| Anti-S-IgG neg^a^, n | 1 (1%) | 1 (1%) |
| Anti-N-IgG pos^b^, n | 84 (77%) | 19 (73%) |
| Anti-N-IgG neg^b^, n | 25 (23%) | 7 (27%) |
| M specific^c^, n | 49 (45%) | 13 (50%) |
| N specific^c^, n | 52 (48%) | 13 (50%) |
| S1 specific^c^, n | 57 (52%) | 17 (65%) |
| S2 specific^c^, n | 56 (51%) | 16 (62%) |
| 4 Epitopes pos, n | 29 (27%) | 11 (42%) |
| ≥1 Epitopes pos, n | 83 (76%) | 21 (81%) |

a Anti-spike IgG antibody; b Anti-nucleocapsid IgG antibody; c specific T cells epitope pool

* Chronic conditions reported by parents in the questionnaire: asthma, hay fever, celiac disease, lactose intolerance, allergies

(other than hay fever), neurodermatitis, diabetes mellitus, inflammatory bowel disease, hypertension, arthritis, other chronic

diseases potentially affecting the immune response (neutropenia, periodic fever with aphthous stomatitis, pharyngitis, and

adenitis (PFAPA) syndrome, renal failure, cystic fibrosis, bronchitis)

**Supplementary Table 2:** Trajectories of the T cell-mediated responses specific to SARS-CoV-2 M, N, S1, and S2 epitope between baseline and follow up according to the participants’ exposure status (see Figure 2).

| Exposure Status  Nov/DEc  2021 | Exposure Status  Jun/JuL  2022 | Baseline  T cell  response | Follow up  T cell  response | M | N | S1 | S2 |
| --- | --- | --- | --- | --- | --- | --- | --- |
| Infected | Hybrid | negative | negative | 6 | 3 | 5 | 5 |
| Infected | Hybrid | negative | positive | 4 | 4 | 4 | 4 |
| Infected | Hybrid | positive | negative | NA | 3 | NA | 1 |
| Infected | Hybrid | positive | positive | 1 | 1 | 2 | 1 |
| Infected | Infected | negative | negative | 18 | 15 | 14 | 19 |
| Infected | Infected | negative | positive | 22 | 15 | 15 | 13 |
| Infected | Infected | positive | negative | 6 | 9 | 9 | 5 |
| Infected | Infected | positive | positive | 9 | 16 | 17 | 18 |
| Negative | Hybrid | negative | negative | 5 | 5 | 4 | 4 |
| Negative | Hybrid | negative | positive | 1 | 1 | 1 | 3 |
| Negative | Hybrid | positive | negative | 1 | 1 | 2 | NA |
| Negative | Hybrid | positive | positive | 1 | 1 | 1 | 1 |
| Negative | Infected | negative | negative | 16 | 13 | 8 | 12 |
| Negative | Infected | negative | positive | 9 | 9 | 9 | 6 |
| Negative | Infected | positive | negative | 2 | 2 | 4 | 3 |
| Negative | Infected | positive | positive | NA | 3 | 6 | 6 |
| Negative | Negative | positive | negative | 1 | 1 | 1 | 1 |
| Negative | Vaccinated | negative | negative | 1 | 1 | 1 | NA |
| Negative | Vaccinated | negative | positive | 2 | 2 | 1 | 4 |
| Negative | Vaccinated | positive | negative | 4 | 4 | 4 | 3 |
| Negative | Vaccinated | positive | positive | NA | NA | 1 | NA |
